# microRNA-106b-5p Promotes Cell Growth and Sensitizes Chemosensitivity to Sorafenib by Targeting the BTG3/Bcl-xL/p27 Signaling Pathway in Hepatocellular Carcinoma

**DOI:** 10.1155/2022/1971559

**Published:** 2022-03-17

**Authors:** Bilegsaikhan Enkhnaran, Guang-Cong Zhang, Ning-Ping Zhang, Hai-Ning Liu, Hao Wu, Shi Xuan, Xiang-Nan Yu, Guang-Qi Song, Xi-Zhong Shen, Ji-Min Zhu, Xiu-Ping Liu, Tao-Tao Liu

**Affiliations:** ^1^Department of Gastroenterology and Hepatology, Zhongshan Hospital, Fudan University, Shanghai 200032, China; ^2^Shanghai Institute of Liver Diseases, Zhongshan Hospital, Fudan University, Shanghai 200032, China; ^3^Key Laboratory of Medical Molecular Virology, Shanghai Medical College of, Fudan University, Shanghai 200032, China; ^4^Department of Pathology, School of Basic Medical Sciences, Fudan University, Shanghai 200032, China

## Abstract

microRNAs (miRNAs) and miRNA-mediated regulatory networks are promising candidates in the prevention and treatment of cancer, but the role of specific miRNAs involved in hepatocellular carcinoma (HCC) remains to be elusive. Herein, we found that miR-106b-5p is upregulated in both HCC patients' tumor tissues and HCC cell lines. The miR-106b-5p expression level was positively correlated with *α*-fetoprotein (AFP), hepatitis B surface antigen (HBsAg), and tumor size. Overexpression of miR-106b-5p promoted cell proliferation, migration, cell cycle G1/S transition, and tumor growth, while decreased miR-106b-5p expression had opposite effects. Mechanistic studies showed that B-cell translocation gene 3 (BTG3), a known antiproliferative protein, was a direct target of miR-106b-5p, whose expression level is inversely correlated with miR-106b-5p expression. Moreover, miR-106b-5p positively regulates cell proliferation in a BTG3-dependent manner, resulting in upregulation of Bcl-xL, cyclin E1, and CDK2, as well as downregulation of p27. More importantly, we also demonstrated that miR-106b-5p enhances the resistance to sorafenib treatment in a BTG3-dependent manner. The in vivo findings showed that mice treated with a miR-106b-5p sponge presented a smaller tumor burden than controls, while the mice injected cells treated with miR-106b-5p had more considerable tumor burden than controls. Altogether, these data suggest that miR-106b-5p promotes cell proliferation and cell cycle and increases HCC cells' resistance to sorafenib through the BTG3/Bcl-xL/p27 signaling pathway.

## 1. Introduction

Hepatocellular carcinoma (HCC), the most prevalent subtype of liver cancer, has become the second leading cause of cancer‐related mortalities worldwide [1, 2]. Most patients progress to unresectable advanced stages due to a lack of accurate noninvasive surveillance, resulting in a dismal survival [3]. Current therapeutic modalities, including approved multitarget inhibitor sorafenib, only slightly improve median survival and hardly result in long-term progression-free survival. Therefore, comprehensive deciphering of the mechanisms underlying rapid relapse and resistance to treatment in HCC remains established.

microRNAs (miRNAs) are small noncoding RNAs that regulate gene expression by directly binding to the 3′-untranslated region (3′-UTR) in targeting mRNAs [4]. Accumulating evidence has shown that miRNAs play important roles in carcinogenesis, lipid metabolism, and chemotherapy resistance by repressing the expression of their targets [5, 6]. Therefore, identifying tumor-associated miRNAs and their target genes is critical for uncovering miRNA mechanisms in tumor progression. Although previous studies have identified miRNAs as essential players of disease progression and resistance to conventional chemotherapies for various cancers, including HCC [7], further investigation is still warranted to unravel the underlying molecular mechanisms between miRNAs and HCC progression.

Our recently published study investigated the miRNA activation role of miR-93-5p in HCC [8]. Our previous study and others also suggested that oncogenic miR-106b-5p, another member of the cluster, links with various types of cancer [9–11], including HCC [12], along with several tumor biological activities, including proliferation, apoptosis, and chemoresistance [13–15]. Chemoresistance constitutes a significant malignant propensity to cancer development and is a substantial obstacle to curing cancer. Recent data indicated changes in miRNA expressions in response to chemotherapy [5, 16].

The purpose of this study is to investigate the significance and underlying mechanisms of miR-106b-5p in cell proliferation and chemotherapy resistance in HCC. Herein, we demonstrate the positive correlations of miR-106b-5p with BTG3 expression in a cohort of HCC tissue and cell lines. Furthermore, we validate that miR-106b-5p promotes G1/S cell-cycle transition and sensitizes HCC cells to sorafenib via the BTG3/Bcl-xL/cyclinE1 pathway.

## 2. Materials and Methods

### 2.1. Cell Lines and HCC Tissues

HCC-LM3, PLC/PRF/5, MHCC-97L, and MHCC-97H were obtained from the Liver Cancer Institute, Zhongshan Hospital of Fudan University (Shanghai, China). A normal liver epithelial cell line (L02) was obtained from the Cell Bank of the Chinese Academy of Sciences (Shanghai, China). All cell lines were maintained in Dulbecco's modified Eagle's medium (DMEM, Corning, Lowell, MA, USA) supplemented with 10% fetal bovine serum (FBS; Corning) and 1% penicillin/streptomycin (Corning). These cells were cultured in a humidified incubator at 37°C with 5% CO_2_.

Sorafenib was purchased from Sigma (Sigma, St. Louis, MO, USA). This compound was dissolved in 100% DMSO (Sigma) and diluted with DMEM to 10 nM for studies. The same concentration of DMSO was added to culture medium as a control. A total of 90 pairs of HCC tissues and adjacent nontumor samples were obtained from patients who underwent surgery at our institution. Written informed consent was acquired from all individuals, and the study was approved by the Institute Ethics Committee.

### 2.2. Cell Transfection

The hsa-miR-106b-5p mimics, hsa-miR-106b-5p inhibitor, and negative controls were attained from RiboBio (Guangzhou, Guangdong, China). The overall sequence of BTG3 was inserted into the pEX-3 vector (GenePharma, Shanghai, China) to construct the BTG3 expression vector. Specific small interfering RNAs (siRNAs) for BTG3 were designed by GenePharma (Shanghai, China). Transfection was performed using INTERFERin (Polyplus, NY, USA). In contrast, transfection of plasmid was performed using a jetPrime reagent (Polyplus, NY, USA) according to the manufacturer's instructions. The miR-106b-5p expression was enhanced by transfection of miR-106b-5p mimics into PLC/PRF/5 and MHCC-97L cells and blocked by transfection of miR-106b-5p inhibitor into MHCC-97H, HCC-LM3 cells. The transfection efficiency was evaluated by real-time quantitative PCR (qPCR).

### 2.3. RNA Extraction and qPCR Analysis

Total RNAs were extracted with a TRIzol reagent (Takara Bio, Kyoto, Japan). For mRNA detection, complementary DNA (cDNA) was synthesized using the miRcute Plus miRNA First-Strand cDNA Synthesis Kit (Tiangen Biotech, Beijing, China). A miRcute Plus miRNA qPCR detection kit (Tiangen) was used for qPCR, with u6 as an internal control. The primers for hsa-miR-106b-5p and u6 were attained from Tiangen Biotech. For mRNA detection, cDNA was synthesized using a PrimeScript RT reagent kit (Takara) and qPCR was performed using the SYBR Premix Ex Taq™ II (Takara).

### 2.4. Fluorescence In Situ Hybridization (FISH) Assay

A specific miR-106b-5p FISH probe was synthesized and applied in the experiment. Hybridization was carried overnight with miR-106b-5p probes according to the manufacturer's instructions of a fluorescence in situ hybridization kit (GenePharma, China). All fluorescence images were captured by a confocal laser-scanning microscope (Leica; Wetzlar, Germany)

### 2.5. Microarrays

Microarray analysis was performed as described before. Briefly, Human miRNA Microarray Release 21.0 (Agilent Technologies, Inc.) was used for screening out the significantly differential expression of miRNAs. *P* < 0.05 and fold-change ≥2.0 were applied for the threshold. Raw data used in the present study are accessible through Gene Expression Omnibus (https://www.ncbi.nlm.nih.gov/geo/query/acc.cgi?acc=GSE108724) [17].

### 2.6. Western Blotting

Total proteins were collected at 48 h after transfection and then electrophoresed using 10–12% SDS-PAGE gels, transferred onto polyvinylidene fluoride membrane (Millipore, Bedford, MA, USA), and blocked with 5% nonfat dry milk in Tris-buffered saline containing 0.05% Tween 20. Membranes were incubated overnight at 4°C with primary antibodies followed by the appropriate secondary antibody for 1 h at room temperature. The signals were detected with electrogenerated chemiluminescence (ECL) developer solution (Millipore). Primary antibodies were presented as follows: anti-*β*-actin (1 : 1000; Cell Signaling Technology, Danvers, MA, USA), anti-Bcl-xL (1 : 1000; CST), anti-BTG3 (1 : 500; Sigma), anti-cyclinE1 (1 : 1000, CST), anti-P27 (1 : 1000, Proteintech, Rosemont, IL, USA), and anti-CDK2 (1 : 1000, Proteintech). The relative optical density of bands was quantified 36 with a GelPro Analyzer (Media Cybernetics, Silver Spring, MD, USA).

### 2.7. Cell Proliferation Assay

According to the manufacturer's protocol, the cell viability was evaluated by a CCK-8 assay (Beyotime). Briefly, cells were seeded on a 96-well plate at a density of 3 × 10^3^ per well containing DMEM (100 mL) in five replicates for each condition and maintained at 37°C with 5% CO_2_. The CCK-8 solution (10 *μ*L) was added to each well and incubated for 2 h after transfected with indicated constructs. The absorbance at 450 nm (with 620 nm as the reference) was measured using a spectrophotometer.

### 2.8. Cell Cycle Analysis

Cell cycle analysis was carried out using flow cytometry with propidium iodide staining (BD Biosciences, Franklin Lakes, NJ, USA). After transfection for 48 hours, the cells were harvested and then fixed with 70% ethanol at 4°C overnight. Next, after washing with PBS three times, the cells were resuspended in 500 *μ*L propidium iodide and incubated at room temperature for 30 min. A total of 10,000 events were counted for each sample and analyzed with a FACScaliber Flow Cytometer (BD Biosciences).

### 2.9. Wound-Healing Assay

Cells were seeded into a 6-well plate. After 12 h of transfection, the cell layer was scratched using a 200 *μ*L pipette tip, and cells were cultured in a DMEM with no FBS. Images of cells were captured at initiation time and 72 h by a microscope (Olympus, Tokyo, Japan). The migration abilities were quantified and normalized by a relative gap distance.

### 2.10. Transwell Assay

The ability of cell to invasion or migration was detected by Transwell (Corning) assay covered with or without Matrigel in the Transwell upper chambers. Briefly, after being transfected for 24 h, cell suspension (1 × 10^4 cells) in 200 *μ*L serum-free DMEM was seeded into the upper chambers, while 500 *μ*L DMEM containing 10% fetal bovine serum was placed in the lower chamber as a chemoattractant. Cells were incubated for 48 h. After removing the nonmigrating or noninvading cells, the remaining cells were fixed with 4% paraformaldehyde, stained with 0.1% crystal violet (Beyotime, Shanghai, China), and then counted under a light microscope (Olympus, Tokyo, Japan) in three random fields per well. The results were expressed as the average number of invasive cells per field.

### 2.11. Luciferase Reporter Assay

Wild-type or mutant of 3′ untranslated region (UTR) of BTG3 was cloned into a PHY-603 luciferase reporter vector (GeneChem, China). Subsequently, miR-106b-5p mimic or negative control combined with BTG3 wild-type or mutant reporter plasmid vector was cotransfected into HEK293T cells. Luciferase activity was detected after transfection for 24 h and tested by an Orion II microplate luminometer (Berthold, Germany).

### 2.12. Immunofluorescence Staining

Cells were inoculated on coverslips in 24-well plates, fixed with 4% paraformaldehyde, permeabilized with 0.2% Triton X-100, and then blocked with 5% bovine serum albumin (Amresco, USA). The cells were then incubated with antibodies against Bcl-xL (CST, 2764) and BTG3 (Sigma-Aldrich, SAB4300958) overnight at 4°C. The secondary antibodies, Alexa Fluor 488 and Alexa Fluor 594 (Yeasen, China), were incubated with the cells at 37°C for 1 h. 4′,6-Diamidino-2-phenylindole (DAPI) was stained to visualize the nucleus. A confocal laser-scanning microscope (Leica; Wetzlar, Germany) was used to capture fluorescence results.

### 2.13. Co-Immunoprecipitation (co-IP) Assay

Co-IP was performed according to the instruction of G-agarose (Millipore, Billerica, MA, USA). Briefly, cells were collected by immunoprecipitation lysis buffer with protease inhibitors. The target protein was immunoprecipitated with the corresponding primary antibodies and evaluated by western blotting. The primary antibodies were as follows: anti-Bcl-xL (CST, 2764) and anti-myc (CST, 2276).

### 2.14. Immunohistochemical Staining

Paraffin slides obtained from in vivo experiments were used for immunohistochemistry. The slides were dewaxed, rehydrated, and subjected to antigen retrieval. Subsequently, the tissues were incubated with a panel of antibodies including anti-Bcl-xL (1 : 300, Cell Signaling Technology, Danvers, USA), anti-BTG3 (1 : 50, Sigma-Aldrich, St. Louis, MO, USA), anti-cyclinE1 (1 : 50, Signalway Antibody, College Park, MD, USA), anti-PCNA (1 : 500, Proteintech, Rosemont, IL, USA), and anti-Ki67 (1 : 4000, Proteintech, Rosemont, IL, USA) overnight. After incubation with the corresponding secondary antibodies, the samples were stained with diaminobenzidine and hematoxylin. Immunohistochemically stained tissue sections were assessed independently by two pathologists.

### 2.15. In Vivo Tumorigenesis in Nude Mice

All experimental procedures were approved by the Institutional Animal Care Committee. Five-week-old male BALB/c nude mice were purchased from the Shanghai SLAC Laboratory Animal (Shanghai, China). Briefly, 1 × 10^6 cells transfected with LV-miR-106b-5p, LV-miR-106b-5p sponge, or LV-miR-NC in 0.2 mL DMEM without FBS were subcutaneously injected into the left flank of the mice (5-week-old, *n* = 4 for each group). Tumors were measured with a caliper every five days. Mice were anesthetized and sacrificed 25 days after tumor inoculation. The tumors were dissected from the body and calculated volume as (length × width^2) × 0.5.

### 2.16. TCGA Data Acquisition

miR-106-5p expression and appropriate survival time in HCC and nontumor tissues were acquired from UCSC Xena (https://xena.ucsc.edu/). Subsequently, the overall survival rate associated with high and low expression of miR-106-5p was analyzed in GraphPad Prism 8.0.2 (GraphPad Prism, La Jolla, CA, USA).

### 2.17. Statistical Analysis

Statistical analysis was performed using the SPSS 22.0 for Window (SPSS, Chicago, USA). Data are presented as the mean ± standard deviation (SD) from at least three independent studies. Student's *t*-test was used to analyze the differences between two groups, and one-way analysis of variance (ANOVA) was used to evaluate the differences between more than two groups. The chi-square test was applied for examining the correlation between miR-106-5p expression and clinicopathological characteristics of HCC patients. Statistical significance was set at *P* < 0.05 (two-sided).

## 3. Results

### 3.1. miR-106b-5p Is Overexpressed in Human HCC Tissues and Cell Lines

**Figure 1:**
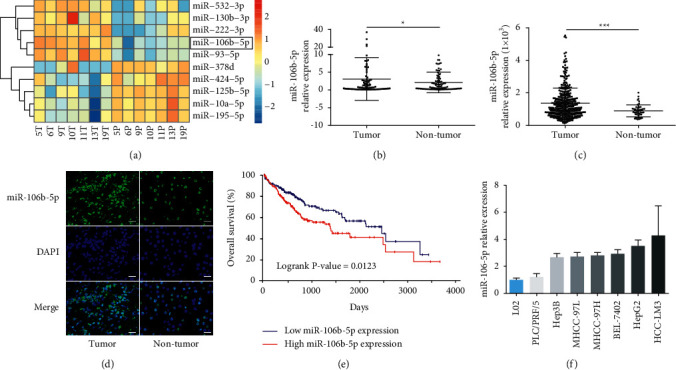
miR-106b-5p is frequently upregulated in HCC tissues and cell lines. (a) A heatmap showing differential expression of miRNAs in 7 paired HCC tissues by miRNA microarray. (b) miR-106b-5p expression in HCC and adjacent para-tumor tissues by qPCR (*n* = 90). (c) miR-106b-5p expression in HCC (*n* = 371) and adjacent para-tumor tissues (*n* = 50) from TCGA database. (d) miR-106b-5p expression in HCC and nontumor tissues by FISH. Scale bar represents 50 *μ*m. (e) Overall survival rate in HCC patients with the low and high miR-106b-5p expression groups. (f) qPCR analysis of miR-106b-5p in HCC cell lines and L02 cells. Data were analyzed using formula 2^−(ΔΔCT)^. Data represent mean ± SD for (b) and (c).  ^*∗*^*P* < 0.05 and ^*∗∗∗*^*P* < 0.001.

Recently, our miRNA expression profiling identified overexpression of miR-106-5p in 7 paired HCC tissues (fold change = 2.46, *P* < 0.001; Figure 1(a)) [17], which is similar to the result of Yen et al. [18]. We examined miR-106b-5p expression in 90 pairs of clinical specimens using qPCR to corroborate this result. In general, the mean expression of miR-106-5p in HCC tissues was 1.45-fold higher than that in matched adjacent tissues (*P*=0.04; Figure 1(b)), which is consistent with the data from the Cancer Genome Atlas (TCGA; Figure 1(c)). Moreover, FISH results showed that miR-106b-5p was significantly increased in HCC tissues compared with matched adjacent tissues and was mainly located in the nucleus (Figure 1(d))

In addition, comparing clinicopathological factors of high and low miR-106b-5p expression groups (Table 1), we found that high miR-106b-5p expression positively correlated with tumor size (*χ*2 = 4.263; *P*=0.039), *α*-fetoprotein (AFP) (*χ*2 = 4.097; *P*=0.043), and hepatitis B surface antigen (HBsAg) (*χ*2 = 9.996; *P*=0.002). Silico analysis of TCGA revealed that high miR-106b-5p expression is negatively associated with the overall survival of HCC patients (Figure 1(e)). To validate the importance of miR-106b-5p in HCC, the expression of miR-106b-5p was next analyzed in a panel of human HCC cell lines, and the normal liver cell line L02 using qPCR. Similarly, the relative expression levels for miR-106b-5p in these seven HCC cells were 1.21- to 4.28-fold compared to that in L02 cells (Figure 1(f)). These results suggest that increased miR-106b-5p is a frequent event in human HCC.

### 3.2. miR-106b-5p Promotes HCC Cell Proliferation and Sorafenib Resistance

**Figure 2:**
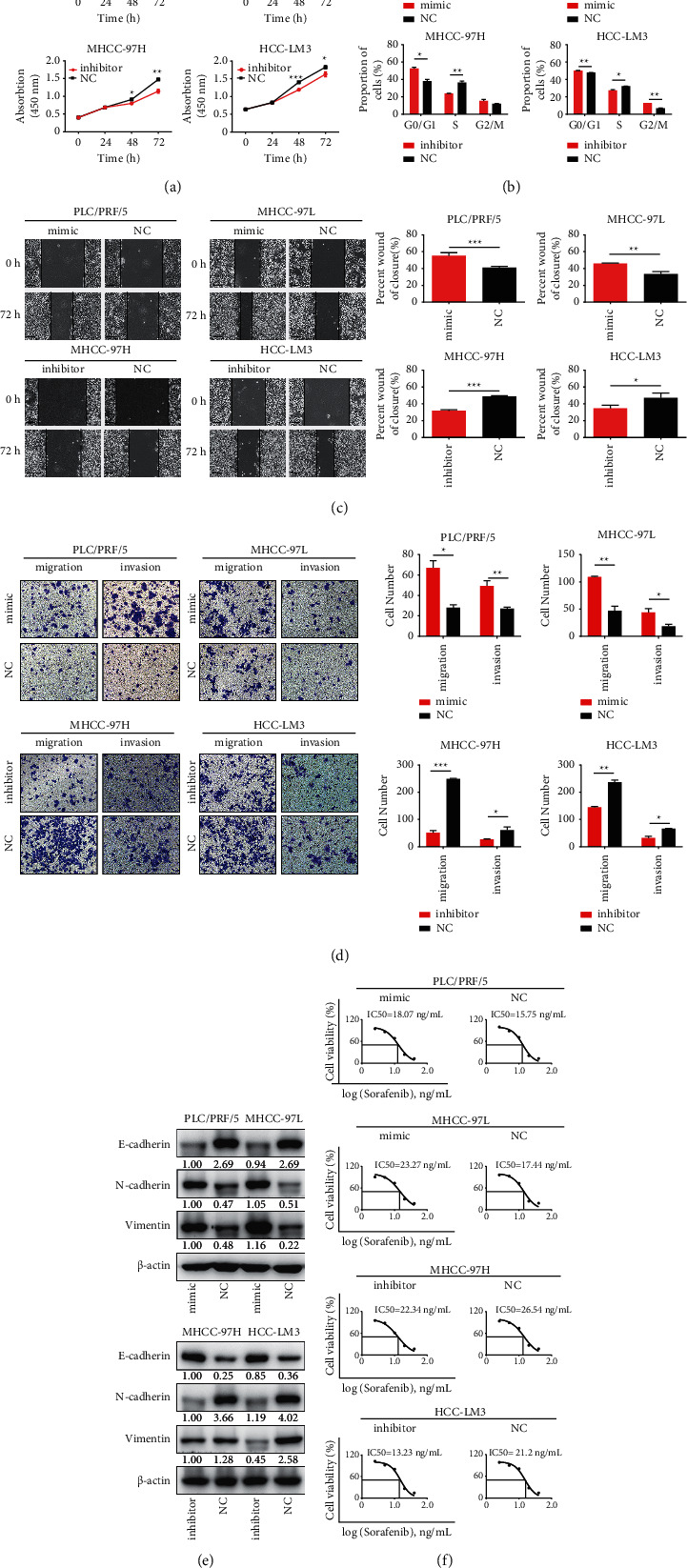
miR-106b-5p promotes HCC cell proliferation, metastasis, and sorafenib resistance. (a) The effect of miR-106b-5p on cell proliferation in HCC cells using CCK8. (b) The effect of miR-106b-5p on the cell cycle of HCC cells using flow cytometry. (c) The effect of miR-106b-5p on the migration of HCC cells using the wound-healing assay. Scale bar, 100 *μ*m. (d) The effect of miR-106b-5p on the invasion of HCC cells using Transwell assay. (e) The effect of miR-106b-5p on the sorafenib resistance in HCC cells using CCK-8. Data represent mean ± s.d. (*n* = 3), NC, negative control.  ^*∗*^*P* < 0.05, ^*∗∗*^*P* < 0.01, and ^*∗∗∗*^*P* < 0.001 by Student's *t*-test.

To gain insight into the biological role of miR-106b-5p in HCC, we performed both inhibition studies using miR-106b-5p inhibitor and overexpression studies using miR-106b-5p mimics (Supplementary Figure 1). First, CCK8 and cell cycle were carried out to evaluate the effect of miR-106b-5p on cell proliferation. Overexpression of miR-106b-5p significantly promoted cell growth and G1/S cell cycle transition in PLC/PRF/5 and MHCC-97L cells. In contrast, suppression of miR-106b-5p markedly inhibited cell proliferation and G1/S cell cycle transition in HCC-LM3 and MHCC-97H cells (Figures 2(a) and 2(b)). Subsequently, wound-healing and Transwell assays were performed to assess the effect of miR-106b-5p on cell migration and invasion, respectively. Overexpression of miR-106b-5p promoted cell migration and invasion in PLC/PRF/5 and MHCC-97L cells. Conversely, suppression of miR-106b-5p exhibited opposite effects in HCC-LM3 and MHCC-97H cells (Figures 2(c) and 2(d)). Furthermore, the expression of E-cadherin/N-cadherin/vimentin serving as metastasis markers was examined. We found that miR-106b-5p overexpression upregulated E-cadherin expression and downregulated N-cadherin/vimentin expression in PLC/PRF/5 and MHCC-97L cells, while miR-106b-5p suppression exhibited opposite effects in HCC-LM3 and MHCC-97H cells (Figure 2(e) and Supplementary Figure 2).

To determine whether miR-106b-5p could influence the sorafenib-induced inhibition of cell proliferation, we next investigated the effect of miR-106b-5p on cell viability in HCC cell lines using a CCK-8 assay. The IC50 of sorafenib following miR-106b-5p mimic treatment increased from 15.75 *μ*M to 18.07 *μ*M in PLC/PRF/5 cells and from 17.44 *μ*M to 23.27 *μ*M in MHCC-97L cells. Moreover, the IC50 for sorafenib following miR-106b-5p inhibitor treatment decreased from 26.54 *μ*M to 22.34 *μ*M in MHCC-97H cells and from 21.2 *μ*M to 13.23 *μ*M in HCC-LM3 cells (Figure 2 (f)). These results suggest that miR-106b-5p promotes cell proliferation and metastasis, along with sorafenib resistance in HCC cells.

### 3.3. miR-106b-5p Directly Targets BTG3

**Figure 3:**
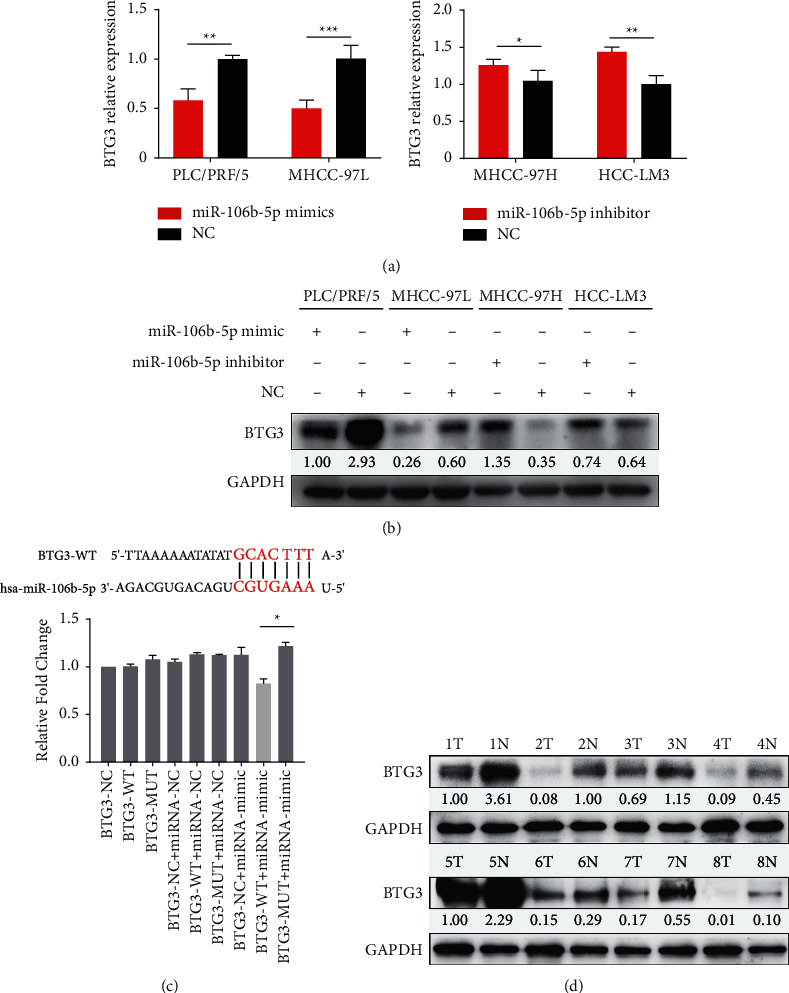
BTG3 is a direct target of miR-106b-5p. The expression of BTG3 in HCC cells after transfected with miR-106b-5p mimic, inhibitor, or its negative control using qPCR (a) and western blotting (b). (c) Sketch of the construction of wild-type or mutant BTG3 3′UTR vectors. (d) Expression of BTG3 in HCC tissues and matched para-tumor tissues from 8 patients. NC, negative control ( ^*∗*^*P* < 0.05).

Considering miRNAs act through inhibition of downstream target genes, we searched for potential target genes of miR-106b-5p in four databases, including miRanda (https://www.microrna.org), DIANA (https://diana.imis.athena-innovation.gr), TargetScan (https://www.targetscan.org), and miRDB (https://www.mirdb.org). Among the searching results, we identified BTG3 as a potential target of miR-106b-5p. In previous studies, BTG3 has been identified as a candidate tumor suppressor gene that plays an important role in tumor growth and metastasis [19–21]. Our findings are consistent with these findings. Overexpressed miR-106b-5p downregulated BTG3 mRNA and protein levels in PLC/PRF/5 and MHCC-97L cells, while downregulated miR-106b-5p elevated BTG3 levels in HCC-LM3 and MHCC-97H cells (Figures 3(a) and 3(b)). Furthermore, a luciferase reporter assay was performed to test the effect of miR-106b-5p on BTG3 in HEK-293T cells expressing wild-type or mutant 3′ UTR BTG3 reporter gene. Twenty-four hours after cotransfection with miR-106b-5p mimic or negative control, the luciferase activities were found to be inhibited in wild-type 3′ UTR reporter gene, rather than the mutant gene by the miR-106b-5p mimic (Figure 3(c)). Moreover, the results from clinical samples revealed that the BTG3 expression was significantly downregulated in tumor tissues compared with adjacent nontumor tissues (Figure 3(d)). Thus, these findings suggest that BTG3 is a direct downstream target of miR-106b-5p.

### 3.4. Effects of BTG3 on HCC Cells

**Figure 4:**
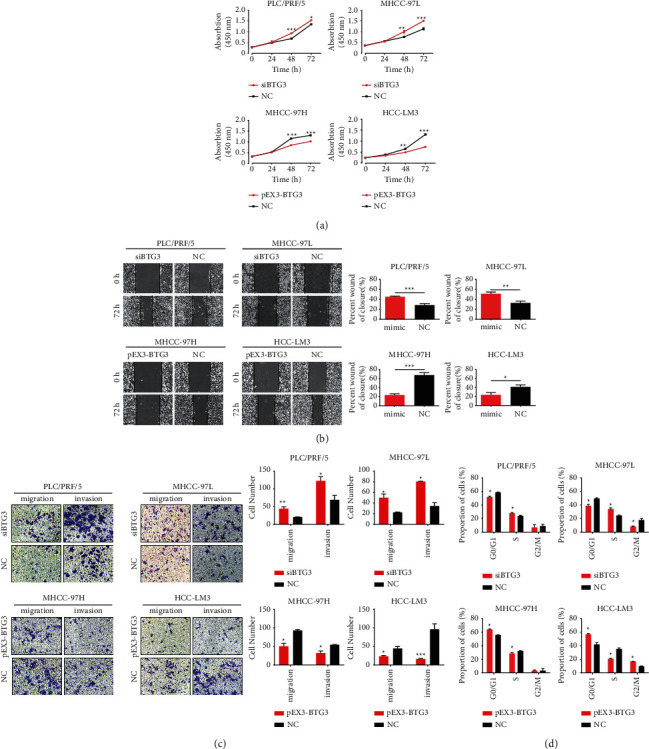
BTG3 suppresses HCC cell proliferation and metastasis. (a) CCK8 measured the effect of BTG3 on cell proliferation in HCC cells. The wound-healing assay (b) and Transwell assay (c) were performed to analyze the effect of BTG3 on the migration and invasion of HCC cells. (d) Flow cytometric assay was performed to analyze the effect of BTG3 on the cell cycle of HCC cells. NC, negative control.  ^*∗*^*P* < 0.05, ^*∗∗*^*P* < 0.01, and ^*∗∗∗*^*P* < 0.001.

To further examine whether BTG3 mediated miR-106b-5p-induced HCC proliferation and metastasis, the pEX3-BTG3 plasmid was constructed to overexpress BTG3, along with BTG3-siRNA being employed to inhibit BTG3 in HCC cells. The CCK8, cell cycle, wound-healing, and Transwell assays were then performed to investigate the function of BTG3 in HCC cell lines. Downregulation of BTG3 simulated the effect of miR-106b-5p mimic and consequently resulted in increased cell proliferation (Figure 4(a)), G1/S transition (Figure 4(d)), and migratory and invasive ability in PLC/PRF/5 and MHCC-97L cells (Figures 4(b) and 4(c)). In contrast, the overexpression of BTG3 also had a similar effect of miR-106b-5p inhibitor and resulted in decreased cell proliferation (Figure 4(a)), G1/S transition (Figure 4(d)), migratory and invasive ability in HCC-LM3 and MHCC-97H cells (Figures 4(b) and 4(c)). These findings suggest that miR-106b-5p enhances HCC cell proliferation and metastasis, at least in part by restraining BTG3 expression.

### 3.5. miR-106b-5p Induces Cell Proliferation and Sorafenib Resistance through the BTG3/Bcl-xL/p27 Signaling Pathway

To determine whether miR-106b-5p promotes HCC proliferation and chemoresistance via inhibition of BTG3, downstream genes of the BTG3 signaling pathway were also analyzed. Based on the results above, we used immunofluorescence staining, co-IP, and western blotting to confirm whether miR-106b-5p and BTG3 affected cell cycle regulatory proteins. Recent studies have demonstrated that TOB, one BTG/TOB family member, is negatively associated with Bcl-xL, a cell cycle-related protein [22, 23]. Thus, we hypothesized that BTG3 might regulate Bcl-xL.

**Figure 5:**
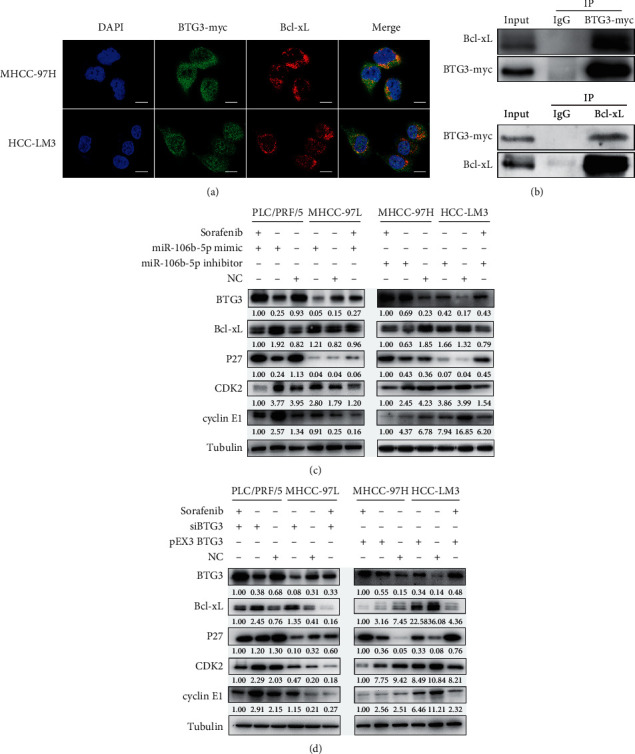
miR-106b-5p induces sorafenib resistance of HCC cells through BTG3/Bcl-xL/p27. (a) Immunofluorescence staining analysis showed the colocalization of BTG3 and Bcl-xL in MHCC-97L and HCC-LM3 cells. Scale bar represents 15 *μ*m. (b) The co-IP study showing the interaction between BTG3 and Bcl-xL in MHCC-97H cells. IgG was used as a negative control. (c) The protein expressions (including BTG3, Bcl-xL, p27, cyclin E1, and CDK2) in HCC cells treated with sorafenib, miR-106b-5p mimic/inhibitor, or sorafenib plus miR-106b-5p mimic/inhibitor for 48 h. (d) Expression of BTG3, Bcl-xL, p27, cyclin E1, and CDK2 was detected after HCC cells incubated with sorafenib, transfected with siBGT3/pEX3-BTG3, or sorafenib plus transfected with siBGT3/pEX3-BTG3 for 48 h NC, negative control.

To test this, we first detected the two proteins' localization in MHCC-97H and HCC-LM3 cells by immunofluorescence staining. BTG3 is mainly colocalized with Bcl-xL in the cytoplasm (Figure 5(a)). Furthermore, the reciprocal co-IP showed that BTG3 and Bcl-xL were seen in the cell lysate. Both proteins were immunoprecipitated from cell lysate in MHCC-97H cells (Figure 5(b)), indicating that BTG3 interacts with Bcl-xL in HCC cells.

Moreover, we found that G1/S-specific protein, including cyclin E1 and CDK2, were increased, while p27 was decreased by miR-106b-5p mimic (Figure 5(c)) and siBTG3 (Figure 5(d)) in PLC/PRF/5 and MHCC-97L cells using western blotting. Subsequently, we analyzed the sorafenib resistance-related protein Bcl-xL. We observed that Bcl-xL expression was increased by miR-106b-5p mimic (Figure 5(c)) and siBTG3 (Figure 5(d)). In contrast, miR-106b-5p inhibitor (Figure 5(c)) and BTG3 overexpression (Figure 5(d)) had an opposite effect on G1/S-specific proteins and sorafenib resistance-related proteins in HCC-LM3 and MHCC-97H cells. Finally, we detected above protein levels in the presence of sorafenib in HCC cell lines. We found that compared to the control group, Bcl-xL, cyclin E1, and CDK2 were decreased, while BTG3 and p27 were increased in all four HCC cell lines (Figures 5(c) and 5(d)). Together, these results indicate that miR-106b-5p induces cell proliferation and sorafenib resistance through the BTG3/Bcl-xL/p27 signaling pathway.

### 3.6. miR-106b-5p Mediates HCC Proliferation In Vivo

**Figure 6:**
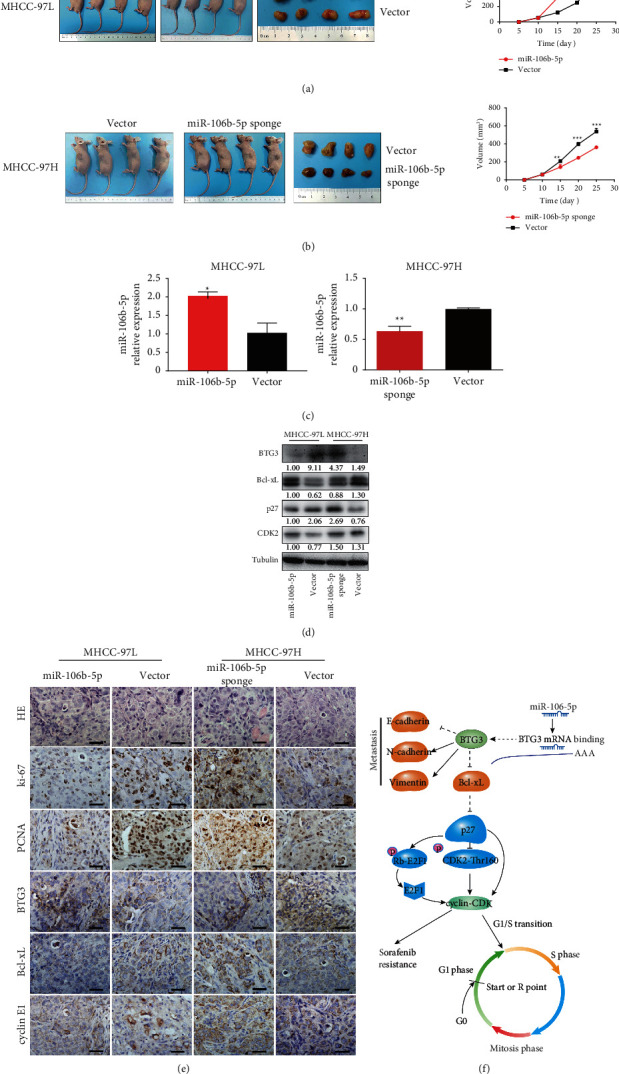
(a, b) In vivo effect of miR-106b-5p. The in vivo effect of miR-106b-5p was evaluated in nude mice, subcutaneously injecting miR-106b-5p, miR-106b-5p sponge, or control cells into nude mice, *n* = 4 per group. Tumor volume was periodically measured for each mouse, and tumor growth curves were plotted. (c) Expression of miR-106b-5p from tumor tissues was validated by qPCR. (d) Expression of BTG3, Bcl-xL, p27, and CDK2 from tumor tissue of nude mice was measured by western blotting. (e) Representative H&E, as well as HC staining for Ki67, PCNA, BTG3, Bcl-xL, and cyclin E1 for tumor tissues. Scale bar represents 30 *μ*m. (f) Schematic diagram illustrating regulatory signaling of miR-106b-5p contributing to proliferation, metastases, and sorafenib resistance in HCC.  ^*∗*^*P* < 0.05, ^*∗∗*^*P* < 0.01, and ^*∗∗∗*^*P* < 0.001.

To verify the phenotype of miR-106b-5p from the in vitro studies, we subcutaneously injected cells treated with miR-106b-5p, miR-106b-5p sponge, or corresponding controls into tumor xenografts. After 25 days of injection, the mice treated with miR-106b-5p sponge presented a smaller tumor burden than controls by IHC (Figures 6(a) and 6(c)) and displayed lower expression for Ki67, proliferating cell nuclear antigen (PCNA), Bcl-xL, and cyclin E1, along with a higher expression for BTG3 in tumor tissues relative to controls by western blotting (Figures 6(d) and 6(e)). In contrast, the mice injected cells treated with miR-106b-5p had more considerable tumor burden than controls (Figures 6(b) and 6(c)) and displayed higher expression for Ki67, PCNA, Bcl-xL, and cyclin E1, along with a lower expression for BTG3 in tumor tissues relative to controls (Figures 6(d) and 6(e)). These results demonstrate that miR-106b-5p promotes HCC proliferation through the BTG3/Bcl-xL/p27 pathway (Figure 6(f)).

## 4. Discussion

Several studies, including our previous work, demonstrated that the aberrant miRNA expression is related to the pathogenesis of HCC [17, 24–27]. However, miRNAs that modulate chemoresistance and their therapeutic potentials remain to be thoroughly elucidated. In this study, we report that miR-106b-5p promotes cell proliferation, cell cycle, and the resistance of HCC cells to sorafenib through the BTG3/Bcl-xL/p27 pathway. We anticipate that these findings, for the first time, will provide novel mechanistic insights into miR-106b-5p-related hepatocarcinogenesis and chemotherapeutic resistance.

miR-106b-5p belongs to the miR-106b/25 cluster. Overexpression of the miRNA-106b-25 cluster was identified in HCC tissues and cell lines, as well as in breast and prostate cancers [28, 29]. Various targets of the cluster have been successively confirmed in HCC and other cancers, and these targets are involved in tumor growth, apoptosis, and metastasis. Substantial evidence suggested that clustered miRNAs cooperatively regulate a similar set of genes belonging to the specific signaling pathways and thus govern biological processes in a coordinated manner. However, in our study, BTG3 is the target of miR-106b-5p, rather than other members of the miR-106b/25 cluster in HCC (data not shown). Given the heterogeneity of HCC, it is possible that the target of the cluster is dependent on the genetic aberrations within the tumor.

Although previous studies reported that miR-106b-5p was involved in the development of HCC [30], no biological activity was observed. Herein, our results suggest that overexpressed miR-106b-5p induces greater tumorigenicity and enhances chemotherapeutic resistance, whereas inhibited miR-106b-5p has opposite effects. Identifications of direct target genes of miRNAs are critical steps for understanding miRNA-related mechanisms of HCC progression. BTG3 is a member of the antiproliferative BTG/Tob (B-cell translocation gene/transducer of ErbB2) protein family, and identified as a tumor suppressor gene involved in the suppression of proliferation, cell cycle progression, apoptosis, and metastasis in various cancers [21, 31, 32]. Based on the dual-luciferase assay and western blotting, our study revealed that BTG3 was a direct downstream target of miR-106b-5p. In vitro loss- or gain-of-function studies demonstrated that BTG3 might function as a tumor suppressor in HCC.

Several studies have demonstrated that the Bcl-2 family influences multiple cellular processes, including cell cycle [33–35]. Followingly, we showed that Bcl-xL, a member of the Bcl-2 family, is downstream of BTG3. The results of immunofluorescence staining and co-IP confirm the existence of protein-protein interaction between BTG3 and Bcl-xL. Since the proteins of the Bcl-2 family are the critical regulator of cellular processes, abnormalities in its function have been implicated in many diseases, including HCC [36]. One study suggested that Bcl-xL-overexpressing HCC patients had significantly shorter disease-free survival after surgery [37]. Other studies indicated that the Bcl-2 family proteins were central components of the sorafenib cytotoxicity in hepatoma cells [38], and Bcl-xL level had been connected to HCC growth and sorafenib-resistance [39, 40].

We then sought to identify whether miR-106b-5p could influence the effects of sorafenib on the viability of HCC cells. Sorafenib is an oral multikinase inhibitor that improved the overall survival of HCC patients, but the clinical response to sorafenib is limited due to resistance [3]. Recent data demonstrated that sorafenib-induced changes in miRNAs [41], while others indicated that several miRNAs were involved in sorafenib resistance in HCC [42, 43]. Of note, it has been suggested that miR-106b-5p participated in drug resistance in multiple cancers [13]. However, whether miR-106b-5p is involved in sorafenib resistance of HCC is still unknown. Our study revealed that miR-106b-5p expression was decreased by sorafenib (data not shown), and its inhibitor sensitized HCC to sorafenib in vitro.

To better understand the mechanisms underlying this effect, cell cycle analysis and western blotting were performed. miR-106b-5p inhibitor blocked G1/S transition; sorafenib potentiated this effect. Whereas, the miR-106b-5p mimic effect was antagonized by sorafenib. We finally found that miR-106b-5p interferes with the expression of BTG3, Bcl-xL, BAX, p27, cyclinE1, and CDK2, possibly hampering the effect of sorafenib. It is well established that cell cycle progression is a predominant factor promoting tumor cell proliferation and inducing chemotherapeutic resistance to sorafenib. Sorafenib can also suppress several critical cell-cycle regulators, including cyclin D, CDKs, and E2F1-Rb-cyclin E1 complex, which plays crucial roles in mediating sorafenib resistance in HCC [44]. In our study, miR-106b-5p induces sorafenib resistance of HCC through Bcl-xL and cyclin E1.

In conclusion, our study showed that miR-106b-5p, as an oncogenic miRNA, was overexpressed in HCC and could promote the proliferation, metastasis, and drug resistance of HCC through activating the BTG3/Bcl-xL/p27 signaling pathway. All these data demonstrate the potential of miRNA therapy, including miR-106b-5p, which could be used in combination with sorafenib to enhance drug sensitivity in HCC.

## Figures and Tables

**Table 1:** Clinical characteristics of selected 75 pairs of HCC and matched nontumor specimens.

Characteristics		miR-106b-5p expression		*χ* ^2^	*P*
		Low	High		
Gender	Male	35	40	0.224	0.636
	Female	6	9		

Age (years)	≤60	17	18	0.210	0.647
	>60	24	31		

Tumor size	≤5 cm	24	18	4.263	0.039
	>5 cm	17	31		

Pathological grade	I-II	27	30	0.206	0.650
	III-IV	14	19		

TNM stage	I-II	33	34	1.446	0.229
	III-IV	8	15		

HBsAg	−	13	3	9.996	0.002
	+	28	46		

AFP	≤200	31	27	4.097	0.043
	>200	10	22		

Vascular invasive	−	29	28	1.775	0.183
	+	12	21		

TNM, tumor-node-metastasis; HBsAg, hepatitis B surface antigen; AFP, *α*-fetoprotein; *χ*^2^, chi-square.

## Data Availability

The data used to support the findings of this study are included within the article.

## Supplementary Materials

Supplementary Materials Supplementary Figure 1: HCC cells transfected with miR-106b-5p mimics, inhibitors, and corresponding negative controls; qPCR analyzed miR-106b-5p levels in indicated cells. NC, negative control.  ^*∗*^*P* < 0.05, ^*∗∗*^*P* < 0.01, and ^*∗∗∗*^*P* < 0.001. Supplementary Figure 2: HCC cells transfected with miR-106b-5p mimics, inhibitors, and corresponding negative controls; qPCR analyzed E-cadherin/N-cadherin/vimentin levels in indicated cells. NC, negative control.  ^*∗*^*P* < 0.05, ^*∗∗*^*P* < 0.01, and ^*∗∗∗*^*P* < 0.001.
