# Supplementary figures and images for: microRNA-106b-5p Promotes Cell Growth and Sensitizes Chemosensitivity to Sorafenib by Targeting the BTG3/Bcl-xL/p27 Signaling Pathway in Hepatocellular Carcinoma
Source: J Oncol. 2022 Mar 17;2022:1971559. doi: 10.1155/2022/1971559 (PMC8947873; doi:10.1155/2022/1971559)

# PLC/PRF/5

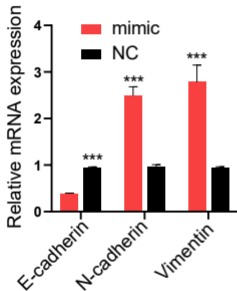

# MHCC-97H

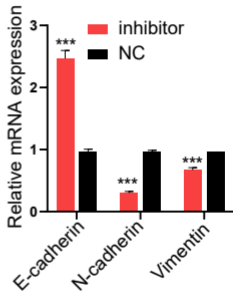

Supplement: Supplementary Materials — Supplementary Figure 1: HCC cells transfected with miR-106b-5p mimics, inhibitors, and corresponding negative controls; qPCR analyzed miR-106b-5p levels in indicated cells. NC, negative control. ∗P < 0.05, ∗∗P < 0.01, and ∗∗∗P < 0.001. Supplementary Figure 2: HCC cells transfected with miR-106b-5p mimics, inhibitors, and corresponding negative controls; qPCR analyzed E-cadherin/N-cadherin/vimentin levels in indicated cells. NC, negative control. ∗P < 0.05, ∗∗P < 0.01, and ∗∗∗P < 0.001. [file 1971559.f1.zip › 1971559.f1/Supplementary Figure 2.pdf]
